# Application of error classification model using indices based on dose distribution for characteristics evaluation of multileaf collimator position errors

**DOI:** 10.1038/s41598-023-35570-1

**Published:** 2023-07-07

**Authors:** Heesoon Sheen, Han-Back Shin, Hojae Kim, Changhwan Kim, Jihun Kim, Jin Sung Kim, Chae-Seon Hong

**Affiliations:** 1 Department of Health Sciences and Technology, Samsung Advanced Institute for Health Sciences and Technology, Sungkyunkwan University, Seoul, South Korea; 2 Department of Radiation Oncology, Gachon University Gil Medical Center, Incheon, South Korea; 3 Department of Radiation Oncology, Yonsei Cancer Center, Yonsei University College of Medicine, Seoul, South Korea; 4 Department of Radiation Oncology, Yonsei Cancer Center, Seoul, South Korea

**Keywords:** Medical research, Oncology, Mathematics and computing, Physics

## Abstract

This study aims to evaluate the specific characteristics of various multileaf collimator (MLC) position errors that are correlated with the indices using dose distribution*.* The dose distribution was investigated using the gamma, structural similarity, and dosiomics indices. Cases from the American Association of Physicists in Medicine Task Group 119 were planned, and systematic and random MLC position errors were simulated. The indices were obtained from distribution maps and statistically significant indices were selected. The final model was determined when all values of the area under the curve, accuracy, precision, sensitivity, and specificity were higher than 0.8 (*p* < 0.05). The dose–volume histogram (DVH) relative percentage difference between the error-free and error datasets was examined to investigate clinical relations. Seven multivariate predictive models were finalized. The common significant dosiomics indices (GLCM Energy and GLRLM_LRHGE) can characterize the MLC position error. In addition, the finalized logistic regression model for MLC position error prediction showed excellent performance with AUC > 0.9. Furthermore, the results of the DVH were related to dosiomics analysis in that it reflects the characteristics of the MLC position error. It was also shown that dosiomics analysis could provide important information on localized dose-distribution differences in addition to DVH information.

## Introduction

Intensity-modulated radiation therapy (IMRT) enables highly conformal and precise dose distribution to the target, reducing the exposure of healthy tissue from unwanted radiation^1,2^. Such remarkable therapeutic outcomes are achieved using a unique beam-shaping device called the multileaf collimator (MLC)^1,3–6^. It is a crucial component of the modulation plan defined for IMRT to generate complex dose distributions^7^. Therefore, regularly monitoring MLC’s accuracy and reproducibility are important to ensure that the actual and planned locations match during treatment^8–10^.

The issue of MLC position uncertainty has been widely investigated for different systems and techniques using radiographic film, ionization chambers, portal imaging devices, and log files^11–18^. However, the utilization of different detectors may yield different results because of their unique characteristics (resolution, detector density, geometric (2D/3D) differences, and calibration method) and potential differences in the gamma calculation method^18–20^.

Gamma analysis^21–23^, developed to quantitatively evaluate the consistency of dose distributions, has been implemented using a combined dose difference (DD) and distance to agreement (DTA) threshold to determine whether each IMRT plan is acceptable in regular clinical practice. However, several groups^4,7,23^ have reported the following limitations in evaluating the uncertainty related to the MLC leaf position: (1) insensitivity to dose errors, (2) absence of correlations with clinical dose errors, (3) difficulty in defining the root cause of the existing discrepancy between dose distributions, (4) differences between the gamma results produced by different measurement devices^24^, and (v) different standard criteria proposed in the Imaging and Radiation Oncology Core (IROC) and TG218 reports^25^. Recently, dosiomics (or radiomics)^1,4,7,26–29^ and structural similarity (SSIM)^4^ have been conducted to determine whether they can be used to overcome the limitations of conventional gamma analysis. First, dosiomics (or radiomics) analysis has been applied to extract valuable features from dose maps between the planned and delivered doses in patient-specific quality assurance (PSQA) by considering the dose or fluence map as an image. Several studies have shown that radiomics-based analysis can effectively detect MLC positioning errors and provide an alternative to conventional gamma analysis^4,5,11,26^. Second, the SSIM index can detect absolute dose errors, gradient discrepancies, and dose structure errors with sub-indices luminance, contrast, and structure^1,4,7,25–27^. Both methods can be employed to describe MLC errors because they show the locations of large discrepancies and different types of error-related patterns. However, no strong indicators or harmonized error characterization or prediction results have been reported because of measurement device bias and patient-specific heterogeneity when using patient data^1,4,7,25–29^. For these reasons, essential research is needed to discover and analyze basic indicators in a state where heterogeneity due to measurement devices and patients is removed before direct application to the clinical practice of various conditions^1,4,7,26–34^.

This study aims to evaluate the characteristics of various MLC position errors correlated with indices using dose distribution, and to use them as reference data for future clinical applications. In this regard, two implementations were preferentially performed as follows: to exclude clinical variability, a single-material AAPM TG-119 phantom was used to establish a treatment plan; MLC position errors were generated in the treatment planning system (TPS) instead of measurements. The difference between the error-free and error dose distributions was analyzed using the gamma index, SSIM index, and dosimetry index to investigate the characteristics of the MLC position error. An index indicating the feature of the MLC position error was found and used to develop an MLC position error prediction model. The dose–volume histogram (DVH) was examined to investigate the clinical relationship. We evaluated the clinical significance by analyzing the final optimal model and DVH parameter results. To the best of our knowledge, this study is the first attempt to analyze the characteristics and predictive models of systematic and random MLC error using dosiomics, gamma, and SSIM indices in the absence of deviation due to clinical, instrumental, and technical differences.

## Materials and methods

### IMRT treatment plans for American Association of Physicists in Medicine^35^ Task Group 119 (TG-119) cases

All plan data were generated and optimized using the segmental MLC (SMLC) mode in the RayStation planning system v5 (RaySearch Laboratories, Stockholm, Sweden). Computed tomography (CT) and structure datasets including phantoms used in this study were downloaded from AAPM TG-119 case^36^. Additionally, AAPM TG-119 suggests the IMRT goals and beam arrangement. For each plan, beam arrangements and planned doses, such as angle and number of beam field on Elekta Versa HD linear accelerator with Agility MLC (Elekta AB, Stockholm, Sweden), were set as recommended by AAPM TG 119 (Supplementary Table S1). The plans were generated using RayStation v5 TPS with the dose calculation algorithm (collapsed cone convolution (CCC)) of a grid size of 2.0 mm for each beam of the static IMRT plans using segmented MLC.

### Simulated MLC leaf position error

**Figure 1:**
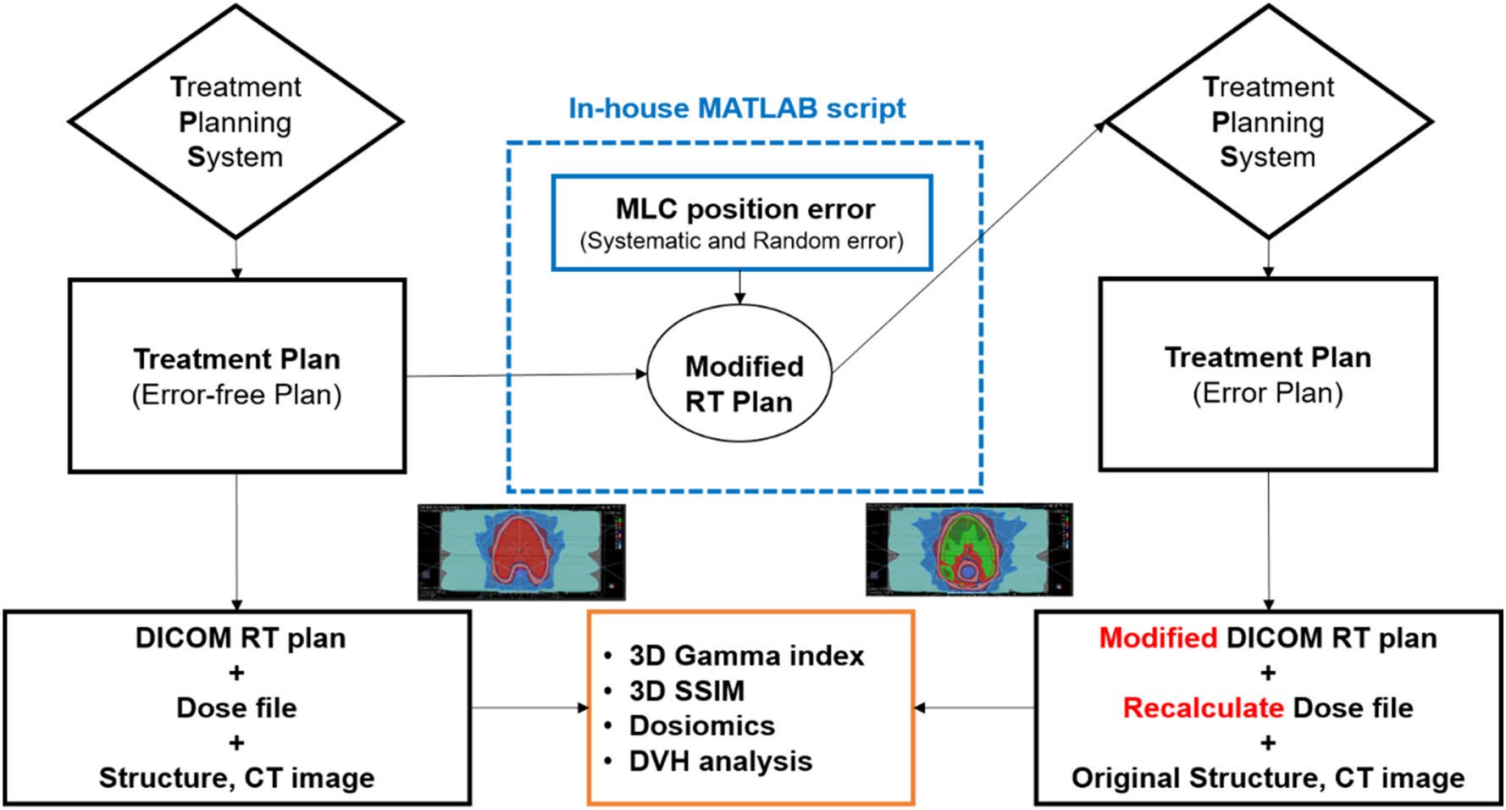
Schematic workflow of simulated systematic and random MLC errors.

This study primarily considered two types of MLC positional errors: systematic error and random error. DICOM-RT plan files were extracted from RayStation v5 to simulate the MLC positional errors. The original MLC position for all the control points described in the DICOM-RT plan file was changed to the locations specified in the DICOM-RT plan file and modified using in-house software(see Supplementary MATLAB source code), developed using MATLAB 2018b (Mathworks, Natick, MA). Subsequently, the modified DICOM-RT plan files were imported back into RayStation v5, as shown in Fig. 1^36^.To evaluate only the effect of the MLC position error, the MU values on all control points between the error-free plan and error plan were not changed, and the same plan objective function was used. Moreover, only recalculation was performed without optimization based on the CCC algorithm.

As shown in Fig. 1, the two types of MLC position errors were induced in each beam of the error-free plan as follows:

For the systematic MLC position error plans, at every control point, the MLC positions surrounding the planning target volume (PTV) were shifted by 0.5 mm, 1.0 mm, 1.5 mm, and 2.0 mm to the right of their original leaf position on one side of the bank.

For the random MLC position error plans, at every control point, the MLC positions in both banks were randomly shifted by a pseudo-random number having a Gaussian distribution of mean value, *μ*, 0.0 mm, 1.0 mm, and 2.0 mm, with a standard deviation, *σ*, 1.0 mm width (1 sigma)^10,37^. When the shift of the leaf position collided with the leaf located on the opposite side bank, it was randomly arranged within the corresponding Gaussian distribution to avoid a collision.

We developed 35 simulated MLC position error plans and total cases for each angle and beam (Table 1).

**Table 1:** MLC position-error types and numbers.

Number of beams	Head and neck	Prostate	C-shape
Error-free	36	28	36
Systematic error	36	28	36
Random error	27	21	27
Total	99	77	99

### Gamma analysis

The gamma-index method evaluates the coincidence between two dose distributions of error-free and error datasets using the percent DD and DTA^38^. We performed a complete local 3D gamma analysis between two dose files for the error-free and error plans with error based on the criteria 3%/3 mm, 3%/2 mm (the AAPM TG-218 recommendation), 2%/2 mm, and 1%/1 mm with a 10% threshold using PTW Verisoft software, version 6.1 (PTW, Freiburg, Germany)^7,10,38,39^. Three-dimensional gamma analysis was conducted for each angle and the entire beam.

### SSIM

The SSIM index is designed to compare and evaluate pairs of images (error-free and error-induced plan) and can be utilized to evaluate the luminance, contrast, and structure of SSIM^4,25,38,40^.

In this study, the entire SSIM index and three subcomponents (luminance, contrast, and structural index) were also evaluated as functions of the beam fields. Preprocessing was not required for SSIM because the size of dose maps was the same. The SSIM index was calculated using MATLAB 2018b (Mathworks, Natick, MA), and it is expressed as follows:

1 $$SSIM \left(x,y\right)={\left[l\left(x,y\right)\right]}^{\alpha }\cdot {\left[c\left(x,y\right)\right]}^{\beta }\cdot {\left[s\left(x,y\right)\right]}^{\gamma }.$$

The default SSIM index is based on the following settings: $\alpha =\beta =\upgamma =1$, and ${C}_{3}= \frac{{C}_{2}}{2}$, and the SSIM index can be calculated as follows:

2 $$SSIM\left(x,y\right)=\frac{(2{\mu }_{x}{\mu }_{y}+{C}_{1})(2{\sigma }_{xy}+{C}_{2})}{({\mu }_{x}^{2}+{\mu }_{y}^{2}+{C}_{1})({\sigma }_{x}^{2}+{\sigma }_{y}^{2}+{C}_{2})},$$

where $l\left(x,y\right)$, $c\left(x,y\right),$ and $s\left(x,y\right)$ are the luminance, contrast, and structure subindex, respectively. ${\mu }_{x}$ and ${\mu }_{y}$, ${\sigma }_{x}$ and ${\sigma }_{y}$, and ${\sigma }_{xy}$ are the local means, standard deviations, and cross-variance for images x and y, respectively.

The specific parameters for the SSIM calculation were set based on previous studies^4,25,40^. The regularization constant is calculated as *C*_*1*_ = (*K*_*1*_*L*)^2^, *C*_*2*_ = (*K*_*2*_*L*)^2^, and *C*_*3*_ = *C*_*2*_/2. *K*_*1*_ and *K*_*2*_ were set to 0.01 and 0.03, respectively, as the default values suggested by Wang et al.^40^. Peng et al.^25^ proposed the default values of *K*_*1*_ and *K*_*2*_ as suitable factors for evaluating the MLC position error in the result of the regularization constant effect according to the *K*_*1*_ and *K*_*2*_ values. The dynamic range, *L*, was set to 200, corresponding to the fraction dose in this study.

### Dosiomics analysis

**Figure 2:**
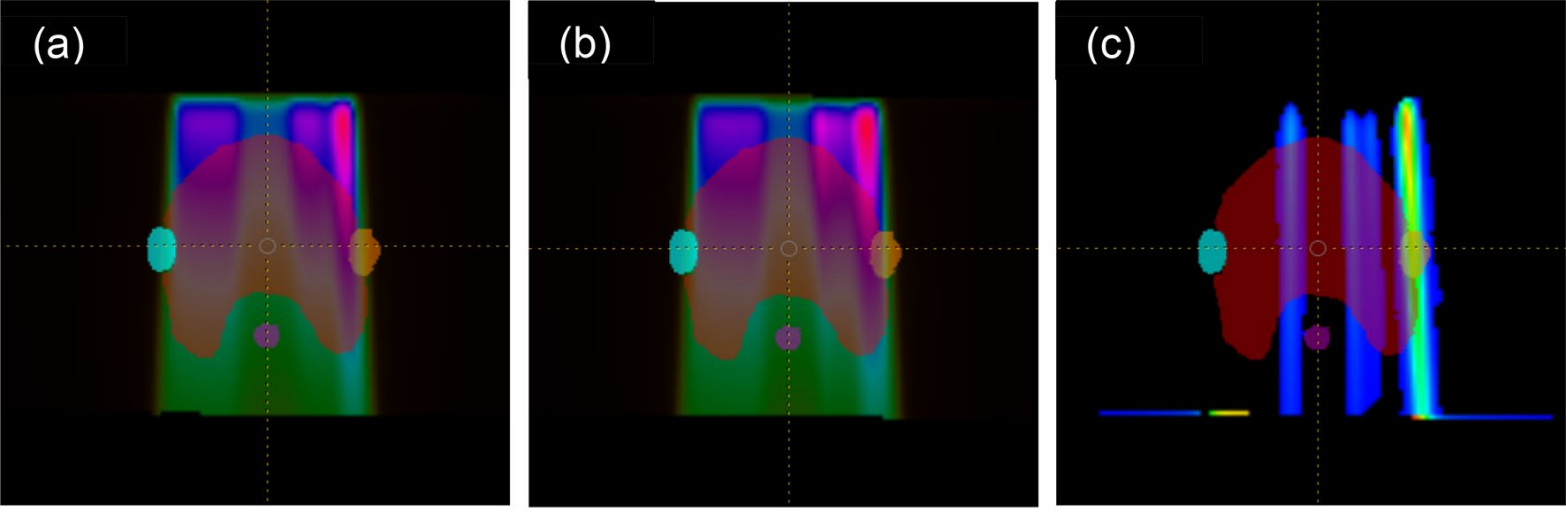
Dose maps. (**a**) Error-free dose map (**b**) Error-induced dose map (**c**) Subtracted (error-induced – error-free) dose map.

For dosiomics analysis, two different dose-distribution datasets were generated: (1) subtracted error-free datasets (simulated error­free dose map—error­free dose map) and (2) subtracted error datasets (simulated error dose map—error-­free dose map) (Fig. 2). The error-­free dose map represents the dose distribution extracted after planning for all control points at the original MLC location via RayStation. To generate a subtracted error-free dose map for dosiomics analysis, two error-free dose maps were required. If the same-dataset error-free dose map (unmodified dose map) is used as a simulated error-free dose map to create sub-error-free, the pixel values will be zero and the radiomics analysis cannot be performed. Therefore, the simulated error-free dose map was generated by systematically moving the error-free dose map by 0.01 mm. The similarity and correlation between simulated error-free and unmodified error-free dose maps were examined using Wilcoxon signed-rank test and Spearman’s rank correlation. Their results showed statistically significant similarity (*p* value > 0.05) in Wilcoxon signed-rank test and the strong correlation (coefficient > 0.97 and *p* value < 0.001) in Spearman's rank correlation (Supplementary Table S2). Therefore, it was confirmed that the simulated error-free had similarity enough to generate sub-error-free when used to run in this experiment. The subtracted dose maps were classified into three types: sub-error-free (simulated error-free dose map—error-free dose map), sub-systematic-error (simulated systematic error dose map—error-free dose map), and sub-random-error (simulated random error dose map—error-free dose map). Class-I included the error-free type and combined systematic-error and random-error. Class-II consisted of error-free and systematic-error types. Class-III comprised error-free and random-error types. Error-type classes in subtracted dose maps are summarized in Table 2.

**Table 2:** Error-type classes in subtracted dose map.

Class of error	Errors
Class-I	Sub-error-free
	Sub-systematic-error + Sub-random-error
Class-II	Sub-error-free
	Sub-systematic-error
Class-III	Sub-error-free
	Sub-random-error

This study performed the dosiomics analysis for Class-I, Class-II, and Class-III. The 275 fluence maps of the four treatment plans exported in the DICOM-RT file from the TPS were analyzed using the Local Image Features Extraction (LIFEx) version 7.1.0 software package (http://www.lifexsoft.org)^41^. For dosiomics index calculations, spatial resampling was 2 mm (X-direction), 2 mm (Y-direction), and 2 mm (Z-direction) in Cartesian coordinates. The size of the bin in intensity discretization was 1. Thirty-four radiomics features were categorized into one conventional feature, two histogram features, and thirty-one texture features^42–44^. Four matrices, namely CLCM, CLRLM, NGLDM, and GLZLM^26,42–44^ were used to determine thirty-one texture features. GLCM was obtained in 13 directions in 3D with one voxel distance relationship between neighboring voxels to indicate the arrangements of pairs of voxels used to calculate textural features. GLRLM was calculated for the 13 different directions to represent the size of homogeneous runs for each gray level. NGLDM was related to the difference in gray levels between one voxel and its 26 neighbors in 3D. GLZLM was calculated directly in 3D to explain the size of homogeneous zones for each gray level. The radiomics features were extracted from the whole subtracted dose map and standardized them to obtain the standard score (z-score) (Supplementary Table S3).

### Statistical analysis

All statistical analyses were performed using RStudio (version 2021.09.1-372; RStudio Software Inc. (Boston, MA, USA)). The error-free data were labeled as “0,” and systematic error data and random error data were labeled as “1.” Gamma, SSIM, and dosiomics indices were examined and selected for developing the MLC position error prediction model. The independence of all the indices of the gamma, SSIM, and dosiomics was investigated to prevent overfitting using Spearman’s rank correlation, backward stepwise elimination, and multicollinearity. First, the indices with Spearman’s rank correlation coefficient higher than 0.8 were removed after the Holm–Bonferroni correction method was applied for all* p* values to correct multiple test comparisons. Then, the remaining indices were filtered by performing backward stepwise elimination. The indices selected through these two steps were selected by the multicollinearity using the variance inflation factor (VIF < 4)^45^. In addition, univariate and multivariate logistic regression were also used for index selection.

### Predictive model development and performance

**Figure 3:**
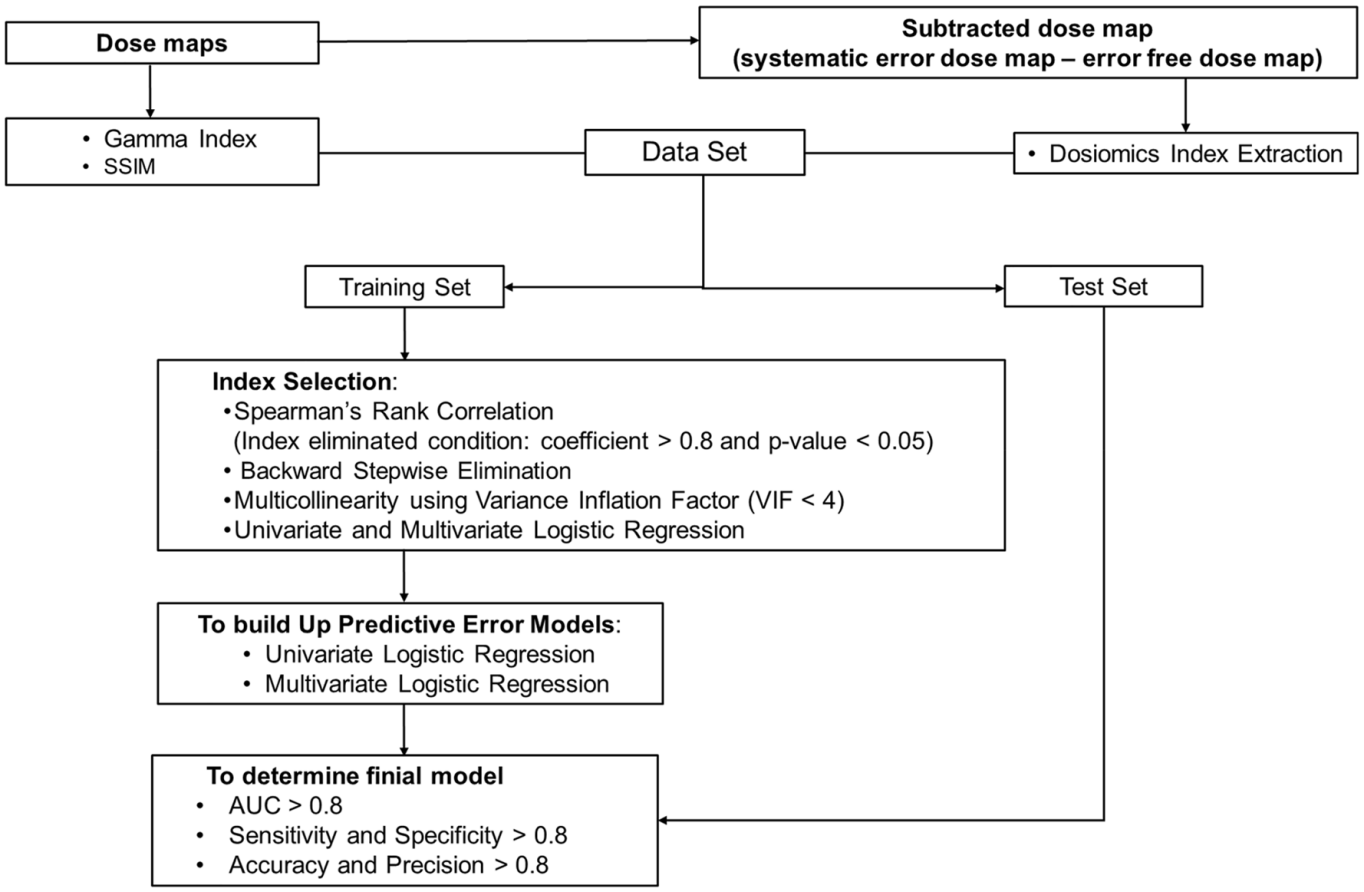
Workflow for developing predictive error models.

Univariate and multivariate logistic regression models for MLC position error prediction was built in RStudio through the following process. To ensure reproducibility of the random sampling, the ‘*set.seed*' function was implemented. The dataset was loaded and split into training and testing sets using the ‘*createDataPartition*’ function. The numbers of training datasets corresponded to 60%–80% of the total number datasets (sub-error-free datasets: 36, sub-systematic-error datasets: 36, sub-random-error datasets: 27) (Supplementary Table S4). The ‘*trainControl*’ function was used to define the training control object, which specified that tenfold cross-validation should be used, repeated 10 times using ‘*repeatedcv*’ method. The ‘*twoClassSummary*’ function was used to summarize the results of the cross-validation process, and ‘*classProbs*’ was set to TRUE to enable probability estimates. The ‘*grid*’ was used to search for the best hyperparameters in the tuning grid. The ‘*train*’ function was then used to train a logistic regression model on the training data using the ‘*glm*’ method and the training control object. The ‘*Accuracy*’ metric was used to evaluate the accuracy of a classification model in the train function. The ‘*predict*’ function was used to predict the class labels for the test data, and the ‘*confusionMatrix*’ and ‘*roc*’ functions were used to evaluate the model in terms of sensitivity, specificity, accuracy, and precision, the area under the curve (AUC) computed based on receiver operating characteristic curve (ROC). The workflow for developing the prediction error model is illustrated in Fig. 3.

### DVH

DVHs for AAPM TG-119^36^ cases with planned IMRT treatment were generated using RayStation v5. The criteria for meaningful differences were underdosed for the PTV and 3% or more for the OAR based on studies on site-based patient quality assurance (QA) standards^46–48^.

## Results

### Gamma and SSIM

**Figure 4:**
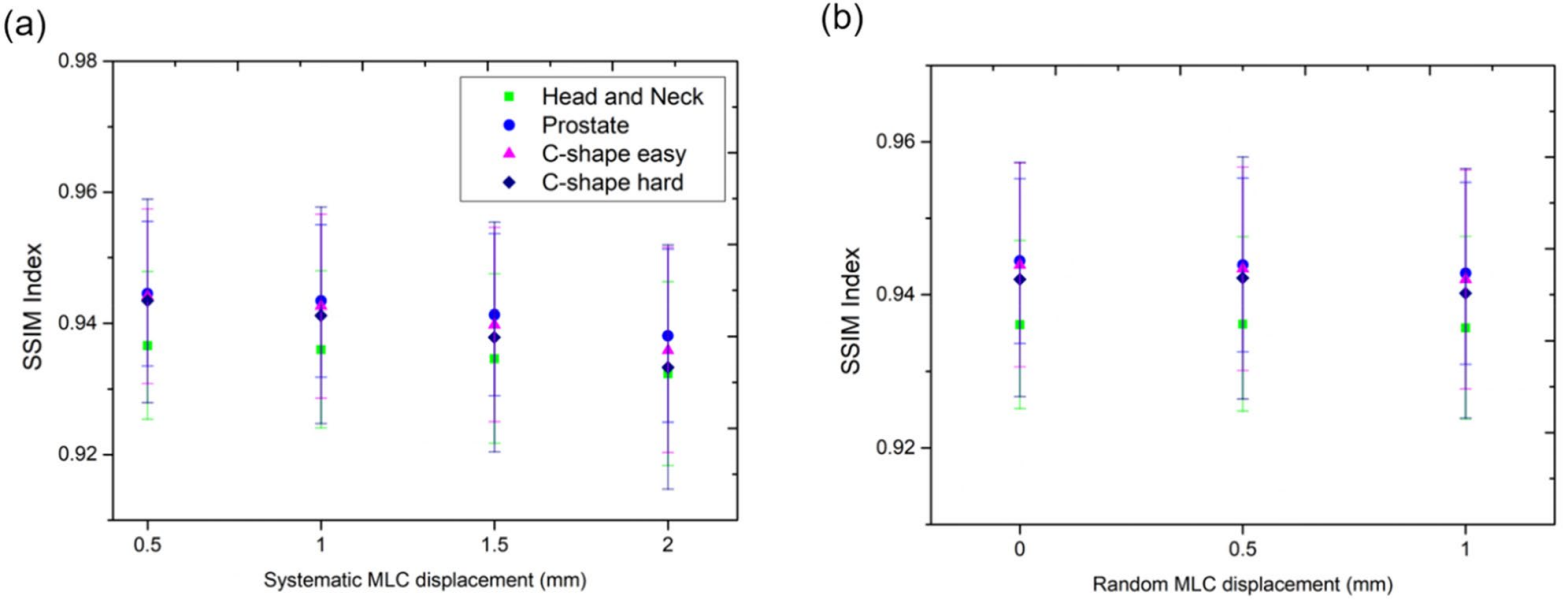
SSIM: (**a**) systematic errors and (**b**) random errors. Y-error bar indicated standard deviation.

The gamma index was analyzed between the error-free and induced error plans, and each documented angle and the total dose in a 3D dose file were analyzed. For the head and neck cases, the gamma-index ranges were 0.926–1.0, 0.848–1.0, 0.758–1.0, and 0.463–0.946 for the 3 mm/%, 2 mm/3%, 2 mm/2%, and 1 mm/1% criteria, respectively. The average SSIM, luminance, contrast, and structure indices were 0.9023–0.9534, 0.9926–0.9995, 0.9983–1.000, and 0.9942–0.9999, respectively. The head and neck cases are summarized in Table 3. The other cases are listed in Supplementary Table S5 (Fig. 4).

**Table 3:** Mean gamma index (2 mm/3%), mean SSIM index, and subcomponent (luminance, contrast, structure) indices for the head and neck cases.

Head and neck	Gamma index	SSIM index	Luminance	Contrast	Structure
Systematic error					
0.5	0.9999	0.9366	0.9982	0.9997	0.9991
1.0	0.9948	0.9360	0.9982	0.9997	0.9991
1.5	0.9591	0.9346	0.9981	0.9997	0.9991
2.0	0.9049	0.9323	0.9980	0.9997	0.9990
Random error					
0.0	1.0000	0.9361	0.9982	0.9997	0.9991
0.5	0.9972	0.9362	0.9982	0.9997	0.9991
1.0	0.9883	0.9357	0.9982	0.9997	0.9991

### Dosiomics analysis

**Figure 5:**
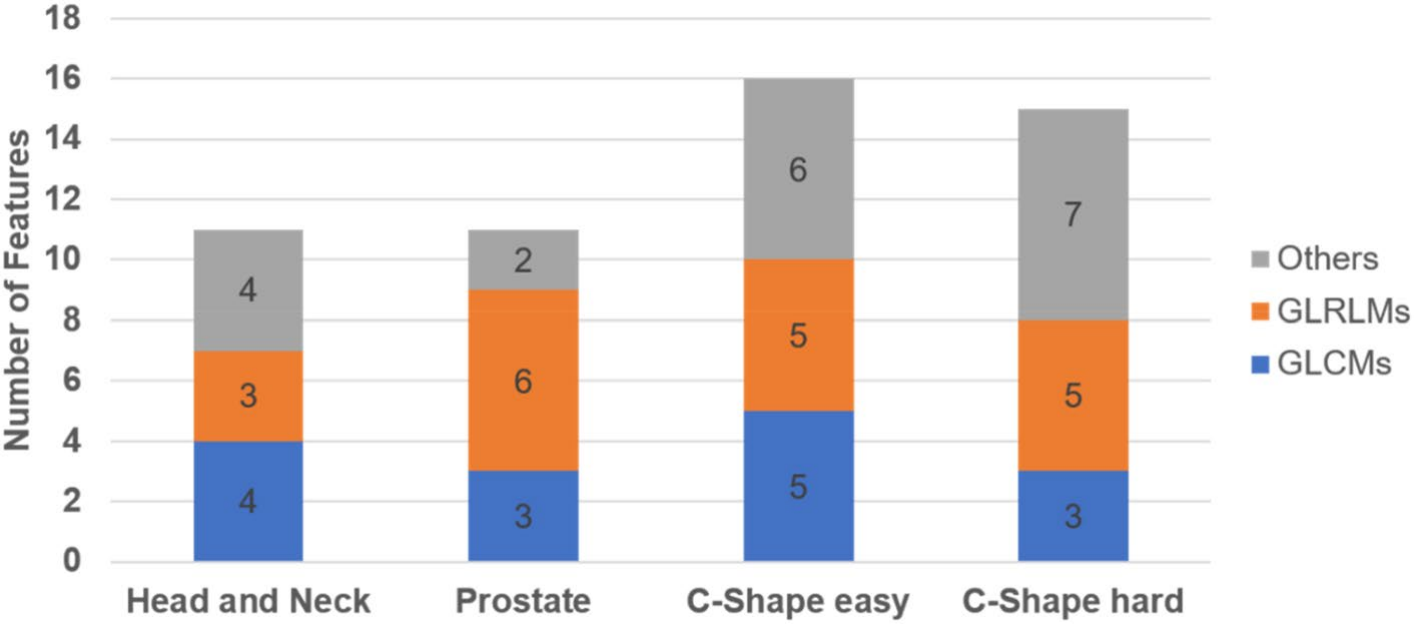
Stacked bar plot showing the distribution of selected features as a function of plan type, with data labels showing the number of features.

The dosiomics indices were extracted from the 275 subtracted dose maps (Class-I, Class-II, and Class-III) generated by all beam fields of the four IMRT plans. Specifically, 34 dosiomics indices were selected through Spearman's rank correlation, backward stepwise elimination, and VIF in all cases. Figure 5 illustrates the number of features according to the plan type. The gray-level run length matrix (GLRLMs) accounted for 35.8% of all features (19/53), and GLCMs approached approximately 28.3%. Eleven, 11, 16, and 15 dosiomics indices were selected from the head and neck, prostate, C-shape easy, and C-shape hard, respectively. The gray-level co-occurrence matrix energy (GLCM_Energy) was the most common dosiomics indices in Class-I and Class-III. Gray-level run length matrix long run high gray-level emphasis (GLRLM_LRHGE) was Class-II's most common dosiomics index.

### Predictive model development and performance

The independent indices of gamma, SSIM, and dosiomics were used to develop predictive models for detecting the MLC position errors after indices were excluded using Spearman’s rank correlation (coefficient > 0.8 and p-value < 0.05), backward stepwise elimination, and VIF (≥ 4) to avoid overfitting among the indices. The independent indices selected using Spearman’s rank correlation and confusion matrix are shown in Supplementary Table S6.

In the final prediction model, only dosiomics indices were selected as significant indices that satisfy the statistical criteria. Therefore, only dosiomics indices were used to develop a dose distribution-based MLC error prediction model. The developed models were evaluated using an independent test dataset. Consequently, the final models were determined when the model’s accuracy, precision, sensitivity, and specificity were 0.8 or more (*p* < 0.05) in Class-I, Class-II, and Class-III. In Class-I, five univariate predictive models (one model each for the head and neck, one for prostate cases, two for the C-shape easy cases, and one for C-shape hard cases) were decided as the final models (Supplementary Table S7). In Class-II, five univariate final predictive models (two models each for the head and neck, one for the prostate, and C-shape easy and hard cases) were determined (Supplementary Table S7). In Class-III, four univariate models were finalized (one for head and neck, prostate, c-shape easy, and C-shape hard cases) (Supplementary Table S7). Six multivariate models were also developed (Supplementary Table S7).

### DVH analysis

The DVHs of the error-free datasets were compared to those of the four systematic error datasets and three random error datasets in terms of clinical effectiveness. In the head and neck case, the relative percentage differences of Sys 1.5 mm, Sys 2.0 mm, and Ran 1.0 mm were greater than 3.0% compared with the DVH of the error-free cases. This indicates that the dose received by the cord and parotids was 3.0% higher. In the prostate case, the dose received by the bladder above the PTV was greater than 3.0% for Sys 1.5 mm, Sys 2.0 mm, and Ran 1.0 mm. The rectum below the PTV received a 3% higher dose than in the error-free case for Sys 1.5 mm, Sys 2.0 mm, and Ran 1.0 mm. In the two C-shape cases, the dose of the cord was 3.0% higher than those in the error-free cases for all error types. The DVH relative percentage difference was almost linear in the systematic error. However, the DVH relative percentage difference of random error showed different tendencies depending on the location of the structure (Supplementary Fig. S1).

## Discussion

In this study, the characteristics of the MLC position error under non-heterogeneous conditions were explained using only the dosiomics indices because they were statistically significant among the gamma, SSIM, and dosiomics indices. The determined dosiomics indices were used for the predictive MLC position error model. The clinical relationship of significant indices and prediction MLC position error model was examined using DVH.

For gamma-index results, even in the case of MLC position systematic 1.0 mm shift, it calculated 0.905 for the C-shape easy case and 0.710 for the C-shape hard case. These results indicate that the dose distribution was affected by plan complexity, and the gamma index was considered to be low. However, there was no significant difference within 3% in the DVH index for PTV and cord. The gamma index had a low correlation with the DVH parameter. The SSIM-index tendency according to the random MLC displacement was relatively small and irregular compared with the SSIM index according to the systematic MLC displacement (Table 3, Fig. 4, and Supplementary Table S5). For the systematic error, only the luminance SSIM subcomponent was more sensitive than the other SSIM subcomponents for the MLC position error. However, its sensitivity was relatively small compared with the dosiomics indices (Supplementary Table S5). For the random error, the gamma and SSIM indices did not convey any trend of the MLC position error.

Among the gamma, SSIM, and dosiomics indices, statistically significant indices representing the characteristics of the MLC position error were extracted from dosiomics. In Class-I, GLCM_Energy was selected as the common significant index for all predictive models. It belongs to the GLCM, representing the dose distribution with co-occurring pixel values at one offset. It was showed that increased error increased the difference between error-free and erroneous data and thus increased non-uniformity of gray level voxel pairs. In Class-II, GLRLM_LRHGE was selected as the common significant index for all cases, and it belongs to the GLRLM, representing the size of the homogenous run. GLRLM_LRHGE shows the distributions with long homogenous runs with high gray levels. As a result, the feature of long homogenous runs with high gray levels was presented for the MLC systematic position error. In Class-III, GLCM_Energy was chosen as the common significant dosiomics index, as in Class-I, except for C-shape hard cases where the significant index was gray-level zone length matrix (GLZLM) gray level_nonuniformity (GLNU). Our result that more than half of the statistically significant indices belonged to the GLCMs was consistent with that of Ma et al*.*^4^*.* In addition, GLRLM_LRHGE that can detect systematic errors, and GLCM_Energy that can detect nonuniformity or random errors, are consistent with the results of the paper published by Landon S. Wootton et al*.*^26^. These two studies were performed using different devices and techniques than ours, and while our study did not include clinical variations, these two studies did include them. Nevertheless, the significant dosiomics indices we found were consistent with those in these studies. These consistent results confirmed that the significant indices we found were a basic index that characterizes the MLC position error regardless of the measurement device, technique, and clinical variation.

The predictive models for the MLC position errors were developed using only dosiomics indices in Class-I, Class-II, and Class-III. The gamma and SSIM indices were disregarded because they were not dedicated adequate weights for developing the predictive models, compared to the dosiomics index. The final error prediction models using the significant dosiomics indices describing the characteristics of the MLC position error exhibited excellent performance with AUC > 0.9, and accuracy, sensitivity, and specificity ≥ 0.8 (p < 0.05), except for the C-shape hard case of Class-III. The more the plan complexity, the more distributed was the influence of the MLC position error; therefore, it was estimated that the accuracy, precision, and specificity values other than AUC and sensitivity values were less than 0.8.

In the DVH analysis, the relative percentage difference in systematic error was observed to increase almost linearly. This indicates that the larger the systematic error in which the MLC position is offset in one direction, the larger is the area where the dose distribution differs. For a random error in which the MLC position is offset in a random direction, the DVH analysis result showed that the relative percentage error increased with different trends depending on the structure’s location. The reason was that the structures in different locations were affected differently for random errors because MLC position errors occur in a random direction. As a result, the systematic error affected the size of the homogenous run in the dose-distribution difference. By contrast, the random error affected the voxel pairs in the dose-distribution difference. These results confirmed that the common significance index indicating the characteristics of the differential dose distribution for systematic errors was GLRLM_LRHGE, and that of random errors was related to GLCM_Energy. Regarding the complexity of the plan, the DVH result showed that the more complex the plan, the greater the relative percentage difference. However, no common significant index representing the dose-distribution difference was found. This is probably because the greater scatter of nonuniformity showing a significant common index as the complexity of the plan increases. These characteristics make different dose distributions depending on the plan complexity, and the significant dosiomics index also varies owing to the resulting random dose-distribution differences. This phenomenon was confirmed in the examples of the C-shape easy case and C-shape hard case. Each of them had two different significant indices, GLCM_Energy and GLZM_GLNU. While GLCM_Energy selected from the C-shape easy case considers a pixel pair with a specific value, the significant index GLZM_GLNU chosen from the C-shape hard case considers the connected voxels. Therefore, depending on the complexity of the plan, the DVH provides information on the dose–volume histogram but cannot show the localized texture differences between dose distributions. In the case of dosiomics analysis, it can provide essential additional information that DVH cannot offer because it can show differences in the local texture of the dose distribution depending on the complexity of the plan.

The results of our study are summarized in three key findings as follows. First, the discovery of principle common significance indices (GLCM_Energy, GLRLM_LRHGE) represents the characteristics of systematic and random MLC position errors and can be used as reference indices for future clinical applications. Second, the prediction MLC position error models developed using statistically significant dosiomics indices showed superior performance (AUC > 0.9). Predictive models have been developed based on specific clinical sites. However, given the significant common indicators found in this study, it is necessary to investigate whether models generated in mixed clinical settings can exhibit excellent predictive power through future studies. Third, the results of DVH in the primary state were confirmed to be related to dosiomics analysis, which represented the characteristics of the dose-distribution difference due to the MLC position error. In addition, it has been demonstrated that in cases with different plan complexity, dosiomics analysis can give more critical information that can be added to the information in the DVH. The results of our study can ultimately provide information related to the characteristics of localized texture using dosiomics analysis. This information could be made more clinically useful by providing additional information to the gamma index and DVH commonly provided by devices and technologies such as log file-based QA or electronic portable imaging devices for dosimetric impact assessment.

Although our results demonstrated the novel clinical findings mentioned above, our study has four limitations. First, the dosiomics index was extracted from the subtracted dose distribution but the gamma and SSIM indices were extracted from dose distributions of the error-free and simulated error instead of their subtracted dose distribution. Second, because only one type of error (MLC position error) was studied under the condition that the factors affecting the error analysis are removed, the direct application of the result to various clinical processes is limited. Third, understanding the relationship between the grid size, preprocessing filter and MLC position error is necessary. In this study, the characterization analysis for the MLC position error uncertainty smaller than the grid size was established by referring to other studies^7,49,50^. Although the MLC position error was smaller than the grid size, there was a difference in the dose distribution; however, the effect of the MLC position error was not fully reflected. Therefore, it is necessary to conduct a study considering the relationship between the grid size and MLC position error. Furthermore, filtered features such as wavelets and Laplacian-of-Gaussian filters were not used as they could potentially introduce noise and artifacts into the dose map which can affect the accuracy and reliability of the results^51^. However, these filters play important roles in dosiomics by emphasizing specific image characteristics, such as edges, and extracting biomarkers^2,51,52^. Therefore, further research is needed to investigate the use of these filters in dosiomics and to develop appropriate methods to mitigate the potential introduction of noise and artifacts. In addition, the features used in this study provide information about the spatial and statistical distribution of dose levels and have been considered sufficient for estimating the MLC error from subtractive dose maps. On the other hand, other studies^1,4^ showed the predictive ability using more dosiomic features. Therefore, it needs to investigate the correlation between the number of dosiomic features and the predictive performance of radiomic models in future studies. Forth, this study used the plan results of static IMRT using segmented MLC to evaluate the characteristics of MLC. However, additional research is needed on how it is reflected when volumetric modulated arc therapy (VMAT) and dynamic MLC, which are clinically applied plan techniques, are applied. In the future, we plan to investigate the detection sensitivity utilizing dosiomics, SSIM, and DVH according to diverse error scenarios and complexities in heterogeneous environments for clinical application.

## Conclusion

The characteristics of the MLC position error were investigated using dose-distribution comparisons of error-free and simulated error datasets. Predictive models were developed and evaluated for the accurate and precise analysis of the MLC position error. Our study highlights three novel results. First, MLC position-error characteristics were described using common significant dosiomics indices (GLCM_Energy, and GLRLM_LRHGE). Second, the finalized logistic regression model for MLC position error prediction showed excellent performance with AUC > 0.9. Third, it was confirmed that the effects of MLC position error on DVH are related to the dosiomics analysis and that the dosiomics analysis provides the important information on localized texture of dose distribution in addition to the DVH information. These clinically significant results are expected to be used as primary data to discover and study clinically meaningful indicators.

## Supplementary Information

Supplementary Information.

## Data Availability

The datasets used and/or analyzed during the current study are available from the corresponding author on reasonable request.
