# Supplementary material for: Application of error classification model using indices based on dose distribution for characteristics evaluation of multileaf collimator position errors
Source: Sci Rep. 2023 Jul 7;13:11027. doi: 10.1038/s41598-023-35570-1 (PMC10328946; doi:10.1038/s41598-023-35570-1)
Supplement: Supplementary file 1 — Supplementary Information. [file 41598_2023_35570_MOESM1_ESM.docx]

**Supplementary information**

**Application of error classification model using indices based on dose distribution for characteristics evaluation of multileaf collimator position errors**

Heesoon Sheen^a,+^, Han-Back Shin^b,+^, Hojae Kim^c^, Changhwan Kim^b^, Jihun Kim^b^, Jin Sung Kim^b^, and Chae-Seon Hong^b,*^

^a^Department of Health Sciences and Technology, Samsung Advanced Institute for Health Sciences & Technology, Sungkyunkwan University

^b^Department of Radiation Oncology, Yonsei Cancer Center, Yonsei University College of Medicine, Seoul, South Korea

^c^Department of Radiation Oncology, Yonsei Cancer Center, Seoul, South Korea

This file contains:

[Supplementary Table S1] IMRT treatment-plan dose goals

[Supplementary Table S2] Results of Wilcoxon signed-rank test and Spearman’s rank correlation

[Supplementary Table S3] Summary of the dosiomics indices

[Supplementary Table S4] Composition ratios of training data sets and test data sets to build up the logistic regression models

[Supplementary Table S5] Mean gamma index (2 mm/3%), mean Structural Similarity Index Measure (SSIM), and subcomponents luminance, contrast, structure index results for the prostate, c-shape easy, and c-shape hard

[Supplementary Table S6] Independent indices remaining after removing the indices with Spearman’s rank correlation coefficients higher than 0.8 (p-value < 0.05) and using confusion matrix

[Supplementary Table S7] Performance of the logistic regression models

[Supplementary Figure S1] DVH Relative percentage difference between error-free and error-simulated data

[Supplementary MATLAB source code]

**[Supplementary Table S1] IMRT treatment-plan dose goals^1^**

| H&N case | Prostate case | C-shape easy case | C-shape hard case |
| --- | --- | --- | --- |
| - PTV   D90 > 5,000  D99 > 4,650  D20 < 5,500   - Cord   Dmax < 4,000   - Parotids   D50 < 2,000 | - PTV   D95 > 7,560  D5 < 8,300   - Rectum   D30 < 7,000  D10 < 7,500   - Bladder   D30 < 7,000  D10 < 7,500 | - PTV   D95 > 5,000  D10 < 5,500   - Cord   D5 < 2,500 | - PTV   D95 ≥ 5,000  D10 < 5,500   - Cord   D5 < 1,000 |

Note: * PTV: radiotherapy planning target volume, *DX: dose received by X% of the volume at X values of 10, 20, 30, 90, 95, 99. *Dmax: maximum received dose of the volume

[Supplementary Table S2] Results of Wilcoxon signed-rank test and Spearman’s rank correlation

| TG119 cases | Wilcoxon signed-rank test  (p - value) | Spearman's rank correlation | |
| --- | --- | --- | --- |
|  |  | coefficient | p - value |
| Head and Neck | 0.39 | > 0.97 | <.0001 |
| Prostate | 0.80 | > 0.97 |  |
| CShape Easy | 0.81 | > 0.98 |  |
| CShape Hard | 0.95 | >0.97 |  |

**[Supplementary Table S3] Summary of the dosiomics indices^36-38^**

| Order of extracted feature | Matrix | Index | Type |
| --- | --- | --- | --- |
| First order | Conventional features | - - Mean dose | Global |
|  | Histogram features | - Skewness, - Kurtosis |  |
| Second order | GLCM  (Gray-Level Co-occurrence based on concurrence Matrix) | - - Homogeneity   - Energy   - Correlation   - Contrast   - Entropy   - Dissimilarity | Regional |
|  |  |  |  |
|  | GLRLM  (Gray-Level Run Length based on voxel-alignment Matrix) | - - SRE (short-run emphasis)   - LRE (long-run emphasis)   - LGRE (low grey-level run emphasis)   - HGRE (high grey-level run emphasis)   - SRLGE (short-run low grey-level emphasis)   - SRHGE (short-run high grey-level emphasis)   - LRLGE (long-run low grey-level)   - LRHGE (long-run high grey-level emphasis)   - GLNUr (grey-level nonuniformity for the run)   - RLNU (run-length nonuniformity)   - RP (run percentage) | Regional |
|  |  |  |  |
| High order | NGLDM  (Neighborhood Gray-Level Different  based on neighborhood intensity-difference Matrix) | - - Coarseness   - Contrast   - Busyness | Local |
|  | GLZLM  (Gray-Level Zone Length based on intensity–size–zone Matrix) | - - SZE (short-zone emphasis)   - LZE (long-zone emphasis)   - LGZE (low grey-level zone emphasis)   - HGZE (high grey-level zone emphasis)   - SZLGE (short-zone low grey-level emphasis)   - SZHGE (short-zone high grey-level emphasis)   - LZLGE (long-zone low grey-level emphasis)   - LZHGE (long-zone high grey-level emphasis)   - GLNUz (grey-level nonuniformity for the zone)   - ZLNU (zone length nonuniformity)   - ZP (zone percentage) | Local |

Note: * Mean dose: mean of dose in the defined volume, *GLCM: Gray-Level Co-occurrence based on the concurrence matrix, *GLNUr: Gray-level nonuniformity for the run, *GLNUz: Gray-level nonuniformity for the zone, *GLRLM: Gray-Level Run Length based on the voxel-alignment Matrix, *LGRE: Low gray-level run emphasis, *LGZE: Low gray-level zone emphasis, *LRE: Long-run emphasis, *LRLGE: Long-run low gray-level, *LRHGE: Long-run high gray-level emphasis, *LZE: Long-zone emphasis, *LZLGE: Long-zone low gray-level emphasis, *LZHGE: Long-zone high gray-level emphasis, *HGRE: High gray-level run emphasis, *HGZE: High gray-level zone emphasis, *MTV: Metabolic tumor volume, *NGLDM: Neighborhood Gray-Level Different based on the neighborhood intensity-difference matrix, *RLNU: Run-length nonuniformity, *RP: Run percentage, *SRE: Short-run emphasis, *SRLGE: Short-run low gray-level emphasis, *SRHGE: Short-run high gray-level emphasis, *ZLNU: Zone length nonuniformity, *ZP: Zone percentage

**[Supplementary Table S4] Composition ratios of training data sets and test data sets to build up the logistic regression models**

*A) Head and Neck*

| Error Class | Matrix Index | Ratio of training data sets | Total data set | | Training data set | | Test data set | |
| --- | --- | --- | --- | --- | --- | --- | --- | --- |
|  |  |  | sub-error-free | sub-error | sub-error-free | sub-error | sub-error-free | sub-error |
| Class-I | GLRLM_RP | 65% | 36 | 63 | 24 | 41 | 12 | 22 |
|  | GLRLM_LRHGE  + GLRLM_GLNU | 70% |  |  | 26 | 45 | 10 | 18 |
| Class-II | GLRLM_RP | 65% | 36 | 36 | 24 | 24 | 12 | 12 |
|  | GLZLM_SZE |  |  |  | 28 | 20 | 8 | 16 |
|  | GLRLM_LRHGE  + GLRLM_GLNU |  |  |  | 23 | 25 | 13 | 11 |
| Class-III | GLCM_Correlation | 65% | 36 | 27 | 25 | 17 | 11 | 10 |

*B) Prostate*

| Error Class | Matrix Index | Ratio of training data sets | Total data set | | Training data set | | Test data set | |
| --- | --- | --- | --- | --- | --- | --- | --- | --- |
|  |  |  | sub-error-free | sub-error | sub-error-free | sub-error | sub-error-free | sub-error |
| Class-I | GLRLM_SRE | 70% | 36 | 63 | 27 | 46 | 9 | 17 |
| Class-II | GLRLM_HGRE | 75% | 36 | 36 | 28 | 26 | 8 | 10 |
| Class-III | GLRLM_SRE | 75% | 36 | 27 | 27 | 20 | 9 | 7 |

*C) C-shape Easy*

| Error Class | Matrix Index | Ratio of training data sets | Total data set | | | | Training data set | | | | | Test data set | | |
| --- | --- | --- | --- | --- | --- | --- | --- | --- | --- | --- | --- | --- | --- | --- |
|  |  |  | sub-error-free | | sub-error | | | sub-error-free | | sub-error | | | sub-error-free | sub-error |
| Class-I | GLCM_Entropy_log10 | 65% | 36 | | 63 | | | 25 | | 40 | | | 11 | 23 |
|  | GLZLM_ZP |  |  |  |  |  |  | 24 | | 41 | | | 12 | 22 |
|  | GLCM_Entropy_log10  + GLZLM_ZP | 80% |  |  |  |  |  | 28 | | 52 | | | 8 | 11 |
| Class-II | GLRLM_LRE | 65% | | *36* | | *36* | | | 24 | | 24 | | 12 | 12 |
|  | GLCM_Energy  + GLRLM_LRHGE | 60% | |  |  |  |  |  | 21 | | 23 | | 15 | 13 |
| Class-III | GLRLM_LRHGE  GLCM_Correlation  + GLRLM_LRHGE  + GLZLM_SZE | 65% | 36 | | 27 | | | 23 | | 19 | | | 13 | 8 |

*D) C-shape Hard*

| Error  Class | Matrix Index | Ratio of training data sets | Total data set | | | Training data set | | | Test data set | | |
| --- | --- | --- | --- | --- | --- | --- | --- | --- | --- | --- | --- |
|  |  |  | sub-error-free | sub-error | sub-error-free | | sub-error | sub-error-free | | sub-error |  |
| Class-I | GLCM_Entropy_log10 | 65% | 36 | 63 | 24 | | 41 | 12 | | 22 |  |
|  | GLCM_Entropy_log10  + GLRLM_GLNU | 70% |  |  | 23 | | 48 | 13 | | 15 |  |
| Class-II | GLCM_Entropy_log10 | 75% | 36 | 36 | 27 | | 27 | 9 | | 9 |  |
| Class-III | GLZLM_GLNU | 65% | 36 | 27 | 23 | | 19 | 13 | | 8 |  |

Note:

- sub-error-free: simulated error-free dose map $-$ error-free dose map
- sub-error: simulated error dose map $-$ error-free dose map
- sub-systematic-error: simulated systematic error dose map $-$ error-free dose map
- sub-random-error: simulated random error dose map $-$ error-free dose map
- Class-I: sub-error-free + sub-systematic-error + sub-random-error
- Class-II: sub-error-free + sub-systematic-error
- Class-III: sub-error-free + sub-random-error

**[Supplementary Table S5] Mean gamma index (2 mm/3%), mean Structural Similarity Index Measure (SSIM), and subcomponents luminance, contrast, structure index results for the prostate, c-shape easy, and c-shape hard**

| Prostate case | | SSIM index | luminance | Contrast | Structure | Gamma index |
| --- | --- | --- | --- | --- | --- | --- |
| Systematic | 0.5 | 0.9445 | 0.9987 | 0.9997 | 0.9992 | 0.993 |
|  | 1.0 | 0.9434 | 0.9986 | 0.9997 | 0.9992 | 0.888 |
|  | 1.5 | 0.9413 | 0.9985 | 0.9997 | 0.9992 | 0.769 |
|  | 2.0 | 0.9381 | 0.9983 | 0.9997 | 0.9991 | 0.645 |
| Random | 0.0 | 0.9444 | 0.9986 | 0.9997 | 0.9992 | 1.000 |
|  | 0.5 | 0.9439 | 0.9986 | 0.9997 | 0.9992 | 0.967 |
|  | 1.0 | 0.9428 | 0.9986 | 0.9997 | 0.9992 | 0.894 |

| C-shape easy | | SSIM index | luminance | Contrast | Structure | Gamma index |
| --- | --- | --- | --- | --- | --- | --- |
| Systematic | 0.5 | 0.9441 | 0.9987 | 0.9998 | 0.9993 | 1.000 |
|  | 1.0 | 0.9426 | 0.9987 | 0.9998 | 0.9993 | 0.905 |
|  | 1.5 | 0.9398 | 0.9985 | 0.9998 | 0.9993 | 0.688 |
|  | 2.0 | 0.9359 | 0.9982 | 0.9998 | 0.9992 | 0.530 |
| Random | 0.0 | 0.9439 | 0.9987 | 0.9998 | 0.9993 | 1.000 |
|  | 0.5 | 0.9434 | 0.9987 | 0.9998 | 0.9993 | 0.989 |
|  | 1.0 | 0.9420 | 0.9986 | 0.9998 | 0.9993 | 0.876 |

| C-shape hard | | SSIM index | luminance | Contrast | Structure | Gamma index |
| --- | --- | --- | --- | --- | --- | --- |
| Systematic | 0.5 | 0.9434 | 0.9998 | 0.9998 | 0.9992 | 0.942 |
|  | 1.0 | 0.9412 | 0.9987 | 0.9998 | 0.9991 | 0.710 |
|  | 1.5 | 0.9379 | 0.9985 | 0.9997 | 0.9990 | 0.611 |
|  | 2.0 | 0.9333 | 0.9983 | 0.9997 | 0.9988 | 0.447 |
| Random | 0.0 | 0.9420 | 0.9987 | 0.9998 | 0.9991 | 0.991 |
|  | 0.5 | 0.9422 | 0.9987 | 0.9998 | 0.9991 | 0.954 |
|  | 1.0 | 0.9402 | 0.9986 | 0.9998 | 0.9991 | 0.728 |

**[Supplementary Table S6]** **Independent indices remaining after removing the indices with Spearman’s rank correlation coefficients higher than 0.8 (p-value < 0.05) and using confusion matrix**

| Error Class | Head and Neck | Prostate | C-shape Easy | C-shape Hard |
| --- | --- | --- | --- | --- |
| Class-I | GLCM_Energy^#^ (GLRLM_GLNU^+^, GLRLM_RP*), GLRLM_LRHGE^+#^ | GLCM_Energy^#^ (GLRLM_SRE*), GLRLM_LRHGE^#^, GLZLM_SZLGE | GLCM_Energy^#^ (GLCM_Entropy_log10*^+^)  GLZLM_ZP*^+^ | Skewness  GLCM_Energy^#^ (GLCM_Entropy_log10*^+^)  GLRLM_GLNU^+^ |
| Class-II | GLCM_Energy^#^ (GLRLM_RP*, GLZLM_GLNU^+^), GLRLM_LRHGE^+#^, GLZLM_SZE*, | GLRLM_HGRE* (GLRLM_LRHGE^#^) | GLCM_Energy^+#^  (GLRLM_LRE*)  GLRLM_LRHGE^+#^ | GLCM_Energy^#^ (GLCM_Entropy_log10*) GLRLM_LRHGE^#^, GLRLM_GLNU |
| Class-III | GLCM_Energy^#^ (GLCM_Correlation*), GLZLM_SZE | GLCM_Energy^#^ (GLRLM_SRE*), GLRLM_LRHGE^#^ | Skewness  GLCM_Energy (GLCM_Correlation^+^)  GLRLM_LRHGE*^+^  GLZLM_SZE^+^ | GLZLM_GLNU* |

Note:

- A (B): B is the index with Spearman’s rank correlation with A (> 0.8 and p-value < 0.05) and is the index used in the final model.
- The * marked index is an index used in the final univariate model.
- The ^+^ marked index is an index used in the final multivariate model.
- The ^#^ marked index is a commonly selected index using Spearman's rank correlation in error classes (Class-I, II, III) and treatment planning sites.

**[Supplementary Table S7] Performance of the logistic regression models**

*A) Head and Neck*

| Error Class | Matrix Index | Accuracy | Precision | Sensitivity | Specificity | AUC |
| --- | --- | --- | --- | --- | --- | --- |
| Class-I | GLRLM_RP | 0.94 | 0.91 | 0.92 | 0.95 | 0.99 |
|  | GLRLM_LRHGE + GLRLM_GLNU | 0.93 | 0.94 | 0.9 | 0.94 | 0.99 |
| Class-II | GLRLM_RP | 0.92 | 0.92 | 0.92 | 0.92 | 0.99 |
|  | GLZLM_SZE | 0.92 | 0.92 | 0.92 | 0.92 | 0.97 |
|  | GLRLM_GLNU + GLRLM_LRHGE | 0.96 | 1 | 1 | 0.92 | 0.97 |
| Class-III | GLCM_Correlation | 0.86 | 0.8 | 0.83 | 0.89 | 0.9 |

*B) Prostate*

| Error Class | Matrix Index | Accuracy | Precision | Sensitivity | Specificity | AUC |
| --- | --- | --- | --- | --- | --- | --- |
| Class-I | GLRLM_SRE | 0.92 | 0.94 | 0.89 | 0.94 | 0.91 |
| Class-II | GLRLM_HGRE | 0.83 | 0.8 | 0.89 | 0.92 | 0.95 |
| Class-III | GLRLM_SRE | 0.88 | 0.86 | 0.89 | 0.86 | 0.9 |

*C) C-shape Easy*

| Error Class | Matrix Index | Accuracy | Precision | Sensitivity | Specificity | AUC |
| --- | --- | --- | --- | --- | --- | --- |
| Class-I | GLCM_Entropy_log10 | 0.91 | 0.95 | 0.83 | 0.95 | 0.96 |
|  | GLZLM_ZP | 0.88 | 0.91 | 0.83 | 0.91 | 0.96 |
|  | GLCM_Entropy_log10 + GLZLM_ZP | 0.84 | 0.91 | 0.86 | 0.83 | 0.94 |
| Class-II | GLRLM_LRE | 0.92 | 0.92 | 0.92 | 0.92 | 0.98 |
|  | GLCM_Energy + GLRLM_LRHGE | 0.96 | 1 | 1 | 0.93 | 1.0 |
| Class-III | GLRLM_LRHGE | 0.86 | 0.88 | 0.92 | 0.78 | 0.92 |
|  | GLCM_Correlation + GLRLM_LRHGE + GLZLM_SZE | 0.95 | 1 | 1 | 0.89 | 0.98 |

*D) C-shape Hard*

| Error Class | Matrix Index | Accuracy | Precision | Sensitivity | Specificity | AUC |
| --- | --- | --- | --- | --- | --- | --- |
| Class-I | GLCM_Entropy_log10 | 0.94 | 0.88 | 0.92 | 0.95 | 0.98 |
|  | GLCM_Entropy_log10 + GLRLM_GLNU | 0.89 | 1 | 1 | 0.83 | 0.94 |
| Class-II | GLCM_Entropy_log10 | 0.92 | 0.92 | 0.92 | 0.92 | 0.99 |
| Class-III | GLZLM_GLNU | 0.76 | 0.75 | 0.83 | 0.67 | 0.92 |

| 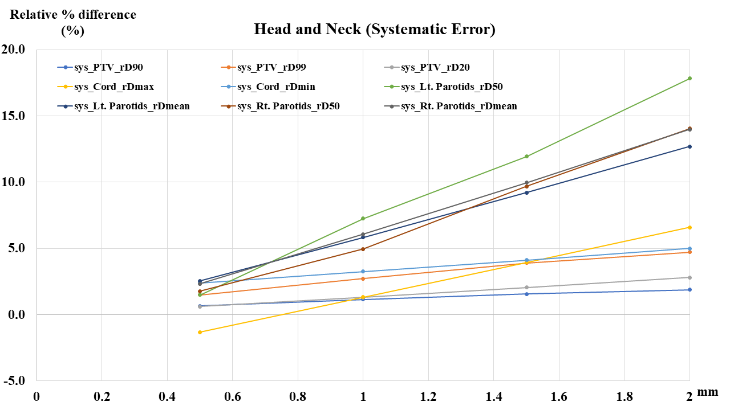(a) | 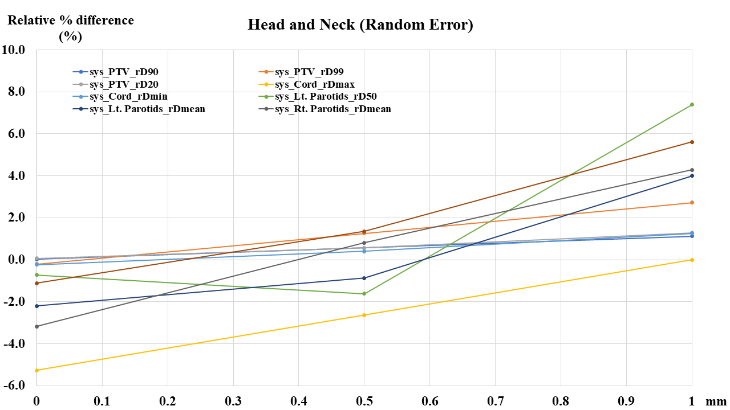(b) |
| --- | --- |
| 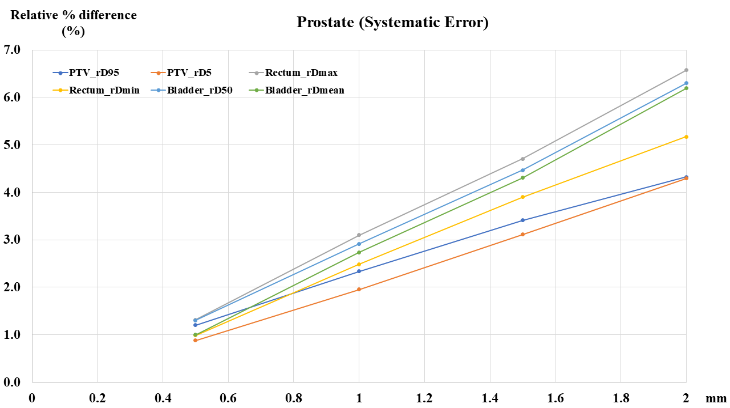(c) | 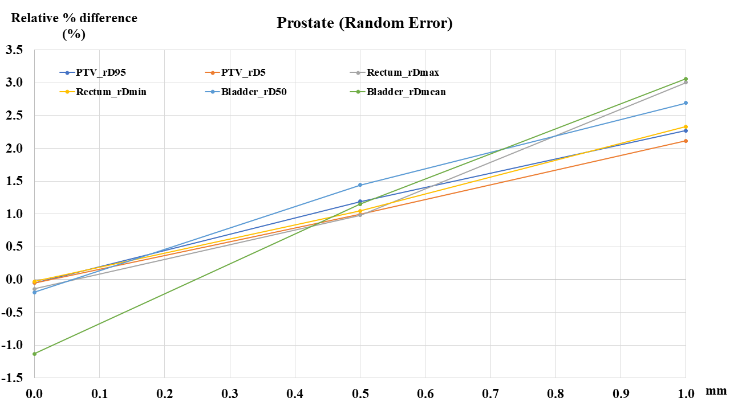(d) |
| 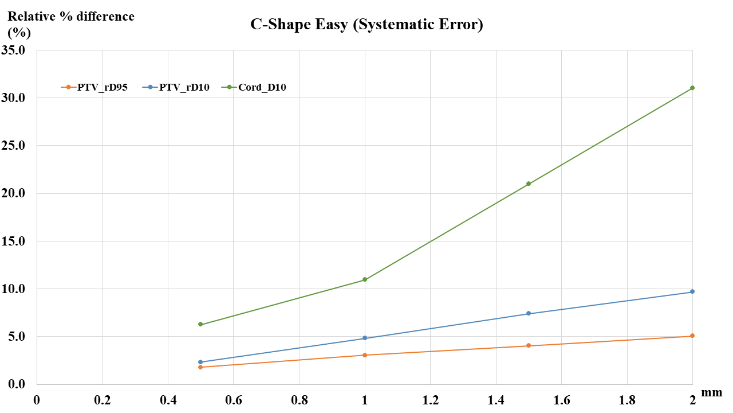(e) | 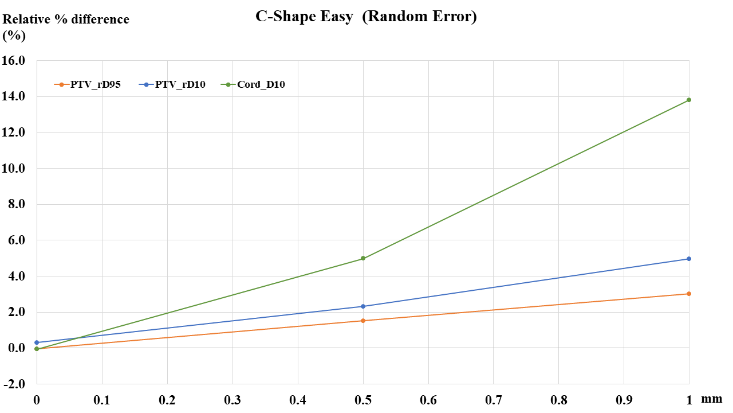(f) |
| 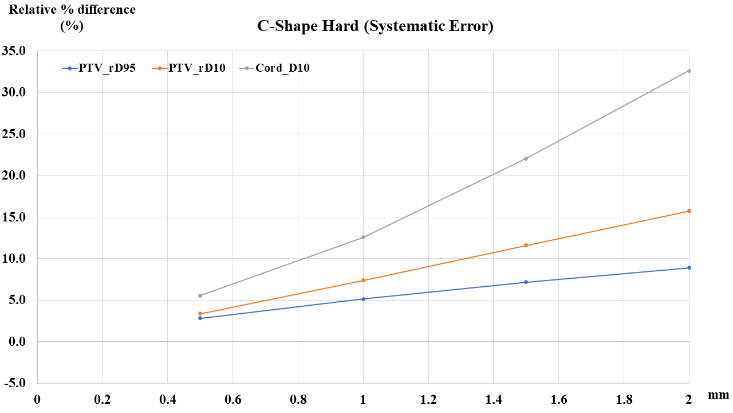(g) | 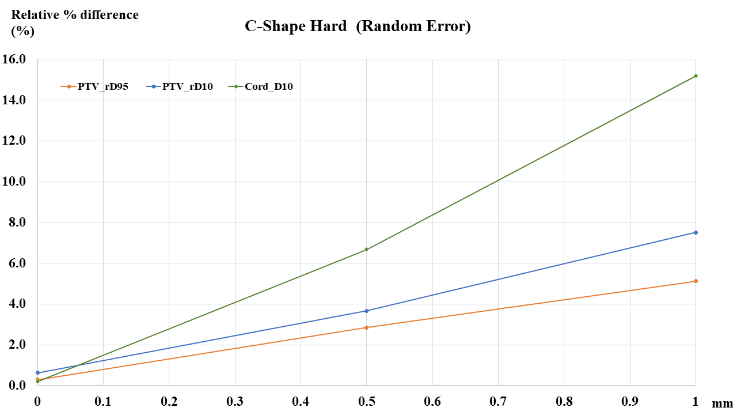(h) |

**[Supplementary Figure S1] DVH Relative percentage difference between error-free and error-simulated data** (a) Head and Neck systematic error, (b) Head and Neck random error, (c) Prostate systematic error, (d) Prostate random error, (e) C-Shape Easy systematic error, (f) C-Shape Easy random error, (g) C-Shape Hard systematic error, and (h) C-Shape Hard random error

Note: * Unit of x-axis in random error is Gaussian distribution of mean value in random error.

[Supplementary MATLAB source code]

The following code shows the programmatically generated MLC shift code using MATLAB 2018b. It is possible to change the MLC shift value in the source code. In this code, it can be confirmed that the MLC shift corresponding to 2.0 mm is applied in the PTV region.

%% Find directory

clc, clear, close all

dirpath = 'Orifinal path';

filelist_phantom = dir([dirpath 'Original.dcm']);

filename_phantom = {filelist_phantom.name};

%% Read original DICOM

temp_phantom_info = dicominfo([dirpath char(filename_phantom)]);

%% MLC CP coordinates

temp_new_info = temp_phantom_info;

num_pair_mlc = 80;

NumofFields = numel(fieldnames(temp_phantom_info.BeamSequence));

NameofFields = fieldnames(temp_phantom_info.BeamSequence);

% MLC Shift value

mlc_shift = 0.20; % MLC shift value(cm)

for k = 1:1:NumofFields

DType = temp_phantom_info.BeamSequence.(char(NameofFields(k))).TreatmentDeliveryType;

NumofCPs = numel(fieldnames(temp_phantom_info.BeamSequence.(char(NameofFields(k))).ControlPointSequence));

NameofCPs = fieldnames(temp_phantom_info.BeamSequence.(char(NameofFields(k))).ControlPointSequence);

MLCposition_total = zeros(num_pair_mlc,2,NumofCPs);

if strcmp(DType, 'TREATMENT')

for j = 1:1:NumofCPs

NumofDPS = numel(fieldnames(temp_phantom_info.BeamSequence.(char(NameofFields(k))).ControlPointSequence.(char(NameofCPs(j))).BeamLimitingDevicePositionSequence));

if (NumofDPS > 2)

Item = 'Item_3';

else

Item = 'Item_2';

end

MLCposition_original = temp_phantom_info.BeamSequence.(char(NameofFields(k))).ControlPointSequence.(char(NameofCPs(j))).BeamLimitingDevicePositionSequence.(char(Item)).LeafJawPositions;

MLCposition_left = MLCposition_original(1:80);

MLCposition_right = MLCposition_original(81:160);

MLCposition_total(:,1,j) = MLCposition_left;

MLCposition_total(:,2,j) = MLCposition_right;

MLCposition_new_left = MLCposition_left;

MLCposition_new_right(1:31,1) = MLCposition_right(1:31);

MLCposition_new_right(32:48,1) = MLCposition_right(32:48) + mlc_shift*10; % PTV region

MLCposition_new_right(49:80,1) = MLCposition_right(49:80);

MLCposition_new_sum = [MLCposition_new_left; MLCposition_new_right];

%mlc coordinate

temp_new_info.BeamSequence.(char(NameofFields(k))).ControlPointSequence.(char(NameofCPs(j))).BeamLimitingDevicePositionSequence.(char(Item)).LeafJawPositions = MLCposition_new_sum;

end

end

end

dicomwrite([],[dirpath 'Multi_Shift_2.0.dcm'],temp_new_info,'CreateMode','copy');

**References**

[1] American Association of Physicists in Medicine. TG-119 IMRT Commissioning Tests Instructions for Planning, Measurement and Analysis. *Med. Phys* **36** 5359-5373 (2009).

[2] Nioche, C. *et al.* Lifex: A freeware for radiomic feature calculation in multimodality imaging to accelerate advances in the characterization of tumor heterogeneity. *Cancer Res.* **78**, 4786–4789 (2018).

[3] Sheen, H. *et al.* Metastasis risk prediction model in osteosarcoma using metabolic imaging phenotypes: A multivariable radiomics model. *PLOS ONE* **14**, e0225242 (2019).
